# Metagenomic Analysis of *Flaviviridae* in Mosquito Viromes Isolated From Yunnan Province in China Reveals Genes From Dengue and Zika Viruses

**DOI:** 10.3389/fcimb.2018.00359

**Published:** 2018-10-24

**Authors:** Pengpeng Xiao, Jicheng Han, Ying Zhang, Chenghui Li, Xiaofang Guo, Shubo Wen, Mingyao Tian, Yiquan Li, Maopeng Wang, Hao Liu, Jingqiang Ren, Hongning Zhou, Huijun Lu, Ningyi Jin

**Affiliations:** ^1^Yanbian University Medical College, Yanji, China; ^2^Institute of Military Veterinary, Academy of Military Medical Sciences, Changchun, China; ^3^College of Veterinary Medicine, College of Animal Science, Jilin University, Changchun, China; ^4^Yunnan Institute of Parasitic Diseases, Simao, China; ^5^Jiangsu Co-innovation Center for Prevention and Control of Important Animal Infectious Diseases and Zoonoses, Yangzhou, China; ^6^Institute of Virology, Wenzhou University, Wenzhou, China; ^7^School of Life Sciences and Engineering, Foshan University, Foshan, China; ^8^Division of Economic Animal Epidemic, Institute of Special Economic Animal and Plant Sciences, Changchun, China

**Keywords:** metagenomic analysis, mosquito, virome, virus detection, phylogenetic analysis

## Abstract

More than 6,000 mosquitoes of six species from six sites were collected and tested for their virome using metagenomics sequencing and bioinformatic analysis. The identified viral sequences belonged to more than 50 viral families. The results were verified by PCR of selected viruses in all mosquitoes, followed by phylogenetic analysis. In the present study, we identified the partial dengue virus (DENV), Zika virus (ZIKV), and Japanese encephalitis virus (JEV) sequences in mosquitoes. Metagenomic analysis and the PCR amplification revealed three DENV sequences, one of which encodes a partial envelope protein. Two ZIKV sequences both encoding partial nonstructural protein 3 and one JEV sequence encoding the complete envelope protein were identified. There was variability in the viral titers of the newly isolated virus JEV-China/YN2016-1 of different passage viruses. The newly identified Zika virus gene from ZIKV-China/YN2016-1 was an Asian genotype and shared the highest nucleotide sequence identity (97.1%) with a ZIKV sequence from Thailand isolated in 2004. Phylogenetic analysis of ZIKV-China/YN2016-1 and ZIKV-China/YN2016-2 with known *Flavivirus* genes indicated that ZIKV has propagated in Yunnan province, China.

## Introduction

Mosquitoes are important hosts and insect vectors for a range of infectious viruses including Japanese encephalitis virus (JEV), Zika virus (ZIKV), dengue virus (DENV), and Getah virus (GETV), which pose a significant threat to human health and represent an economic burden worldwide (Pham et al., 2005; Klungthong et al., 2007). Mosquitoes acquire viruses when consuming blood from hosts undergoing viremia. The viruses then replicate and propagate in the insect host before being introduced into further victims during biting and blood feeding (Ritchie et al., 2007; Motooka et al., 2015). Mosquitoes feed on a wide range of hosts including humans and other vertebrates, invertebrates, and plants (Shi et al., 2014). Yunnan province in China harbors a diverse range of mosquito-borne viruses (Feng et al., 2015); therefore, regional surveillance is imperative. The detection of viruses in mosquitoes is usually performed by using reverse transcription polymerase chain reaction (RT-PCR) and nested PCR approaches (Almeida et al., 2005; Houghton, 2009; Li et al., 2015; Sim et al., 2015). However, compared with Illumina sequencing (Alquezar-Planas et al., 2013; Cholleti et al., 2016; Ergünay et al., 2017), these traditional methods are time-consuming and labor-intensive to detect low-level viromes in mosquito vectors (He et al., 2013; Miesen et al., 2016). Therefore, metagenomic analysis of mosquitoes is likely to be of great value to avoid missing the detection of viruses with high infectivity and pathogenicity as well as the detection of previously unknown viruses.

The present study aimed to build a valid surveillance method to monitor the distribution of Flaviviridae from mosquitoes in Yunnan province, China and to provide useful insights into viral isolation, prevention, and control. Diverse and abundant viromes from mosquitoes isolated in Yunnan province were investigated using metagenomic sequencing and nested PCR. The presence of DENV, JEV, and ZIKV viruses was confirmed, and JEV was isolated. This preliminary exploration of the metagenomes of mosquito-borne viruses lays the foundation for further research on the territorial distribution, diversity, and surveillance of mosquito-borne viruses in China and other countries.

## Materials and methods

### Mosquito collection

**Figure 1:**
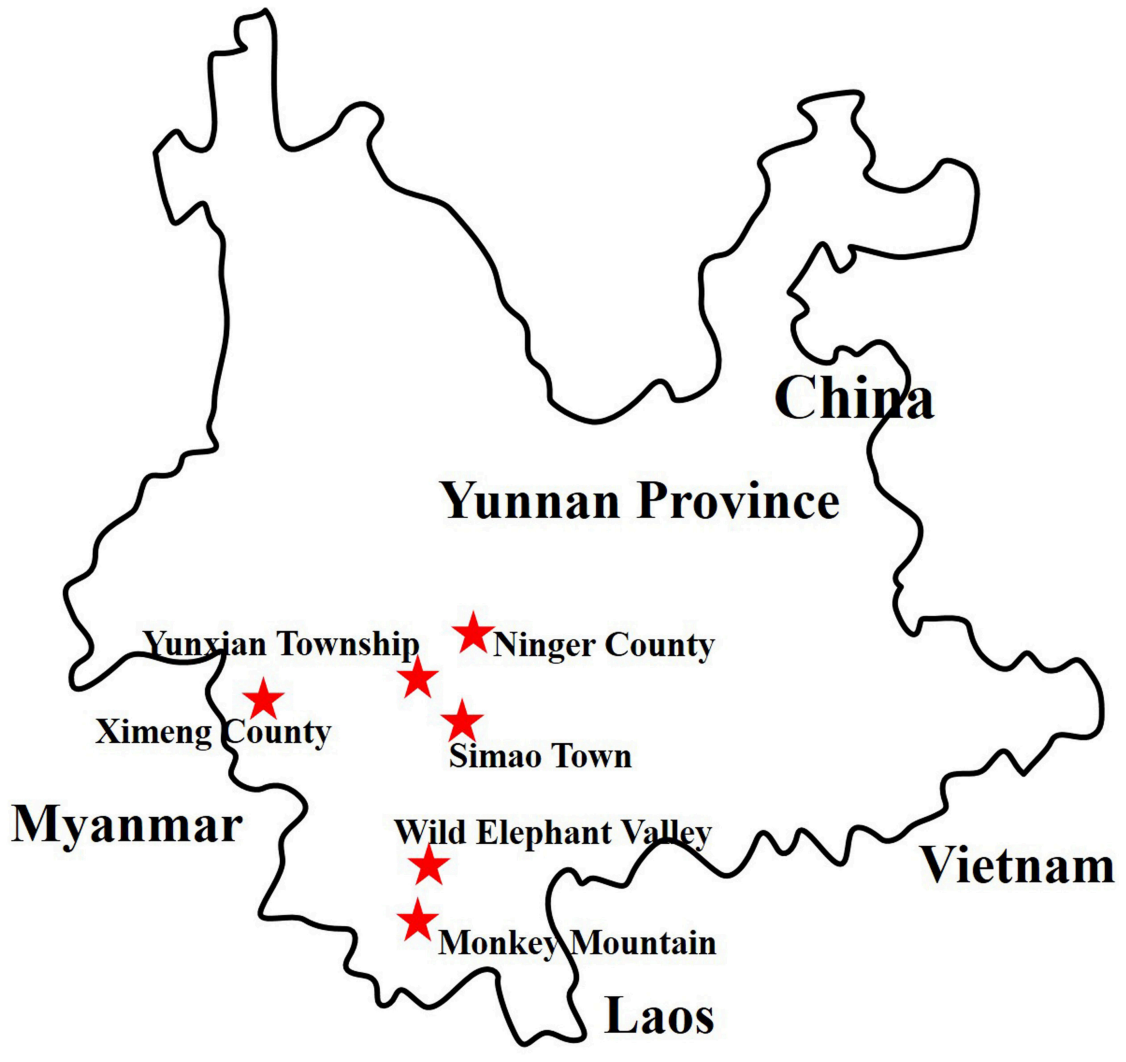
Distribution of sample collection sites in Yunnan province, China, 2016. The sample collection sites are labeled with red stars.

More than 6,000 living or freshly dead female mosquitoes were collected in Yunnan province, China, during August and September 2016 (Figure 1). Sample I comprised *Culex tritaeniorhynchus* (*C. tritaeniorhynchus*). Sample II was a mixture of *Armigeres obturbans* (*Ar. obturbans*) and *Aedes albopictus* (*Ae. albopictus*). Sample III was a collection of *Anopheles sinensis* (*An. sinensis*), *Uranotaenia hebes* (*U. hebes*), and *Armigeres durhami* (*Ar. durhami*) (Table 1). The mosquito samples were grouped based on species. *C. tritaeniorhynchus* was the predominant transmitting vector of JEV, and *Ar. obturbans* and *Ae. albopictus* were the predominant transmitting vectors of DENV and ZIKV (Byrd et al., 2013). The identified species of mosquitoes were collected separately and stored at −80°C. This research was approved by the Ethics Committee and the Research Ethics Committee of Yanbian University Medical College. All experiments involving active viruses were performed in a biosafety level 3 laboratory.

**Table 1:** Mosquito samples used in the metagenomic analysis and data from Illumina sequencing.

**Sample**	**Species**	**Number**	**Location**	**Total reads number**	**Average (nt)**	**Viral reads**	**Viral contigs**
Sample I	*Culex tritaeniorhynchus*	4,400	Yunnan province^*^	7,486,421	187	1,272,692	6,325
Sample II	*Armigeres obturbans*	600	Yunnan province^*^	7,908,381	187	18,980	1,426
	*Aedes albopictus*	300	Yunnan province^*^				
Sample III	*Anopheles sinensis*	300	Yunnan province^*^	7,569,503	184	1,048,376	5,894
	*Uranotaenia hebes*	200	Yunnan province^*^				
	*Armigeres durhami*	300	Yunnan province^*^				
Total/Average				22,964,305	186	2,340,048	13,645

* *Collection sites (Figure 1) were Monkey Mountain (N 22° 0′, E 100° 50′) and Wild Elephant Valley (N 22° 10′, E 100° 51′) in Jinghong city, and Yunxian township (N 22° 50′, E 100° 46′), Simao town (N 22° 47′, E 100° 58′), Ximeng county (N 22° 38′, E 99° 35′), and Ninger county (N 23° 03′, E 101° 02′) in Puer city*.

### Preparation of mosquito samples

Briefly, each mosquito sample was mixed and ground using a tissue grinder with SM buffer (50 mM Tris, 10 mM MgSO4, 0.1 M NaCl, pH 7.5) (MeilunBio, Dalian, China). To remove mosquito debris and other materials, the mixed samples were centrifuged at 13,000 × g for 20 min, and the supernatants were used to extract the viral nucleic acid.

### Extraction of viral nucleic acid and reverse transcription

To remove the free nucleic acid and eliminate the contaminating host genomic DNA, 14 U Turbo DNase (Ambion, Austin, TX, USA), 25 U Benzonase Nuclease (Novagen, San Diego, CA, USA), 20 U RNase I (Fermentas, Ontario, Canada), and 10 × DNase buffer (Ambion) were added to 127 μL of the supernatants to a final volume of 150 μL, followed by digestion at 37°C for 1 h. The viral nucleic acid in the obtained products was extracted using a virus nucleic acid isolation kit (Bioer Technology, Hangzhou, China) according to the manufacturer's instructions. The total viral nucleic acids were reverse-transcribed using anchored random primers and Superscript III reverse transcriptase (Invitrogen, Carlsbad, CA, USA). The anchored random primers (Table 2) were added separately to the viral nucleic acid and incubated at 75°C for 5 min and then placed on ice for 5 min for denaturation. To obtain the reverse-transcribed product, 40 U of RNase OUT (Invitrogen, Carlsbad, USA), 200 U of SuperScript III reverse transcriptase (Invitrogen), 1 μL of 0.1 M dithiothreitol (DTT) (Invitrogen), 1 μL of 10 mM dNTPs (TaKaRa, Dalian China), 4 μL of 5 × first-strand buffer (Invitrogen), and RNase-free H_2_O (TaKaRa) were added to a final volume of 20 μL and incubated at 25°C for 10 min, followed by 50°C for 60 min and then 75°C for 10 min.

**Table 2:** Barcode DNA used in the metagenomic analysis (He et al., 2013).

**Primer type**	**Primer number**	**Primers (5′-3′)**
Anchored random primers	RT1	GCCGGAGCTCTGCAGATATCNNNNNN
	RT 2	GTATCGCTGGACACTGGACCNNNNNN
	RT 3	ATCGTCGTCGTAGGCTGCTCNNNNNN
	RT 4	CGTAGATAAGCGGTCGGCTCNNNNNN
	RT 5	CATCACATAGGCGTCCGCTGNNNNNN
	RT 6	CGCAGGACCTCTGATACAGGNNNNNN
	RT 7	CGTCCAGGCACAATCCAGTCNNNNNN
	RT 8	CCGAGGTTCAAGCGAGGTTGNNNNNN
	RT 9	ACGGTGTGTTACCGACGTCCNNNNNN
	RT 10	CGACCCTCTTATCGTGACGGNNNNNN
	RT 11	GAGCCCCTAGACACAACGACNNNNNN
	RT 12	GGTGGGCGTGTGAAATCGACNNNNNN
	RT 13	GAAAATGAGAGGGGAGGCGGNNNNNN
Barcode primers	Primer1	GCCGGAGCTCTGCAGATATC
	Primer2	GTATCGCTGGACACTGGACC
	Primer3	ATCGTCGTCGTAGGCTGCTC
	Primer4	CGTAGATAAGCGGTCGGCTC
	Primer5	CATCACATAGGCGTCCGCTG
	Primer6	CGCAGGACCTCTGATACAGG
	Primer7	CGTCCAGGCACAATCCAGTC
	Primer8	CCGAGGTTCAAGCGAGGTTG
	Primer9	ACGGTGTGTTACCGACGTCC
	Primer10	CGACCCTCTTATCGTGACGG
	Primer11	GAGCCCCTAGACACAACGAC
	Primer12	GGTGGGCGTGTGAAATCGAC
	Primer13	GAAAATGAGAGGGGAGGCGG

### Synthesis of double-strand cDNA (dscDNA)

Before the synthesis of dscDNA, 1 μL of RNase H (TaKaRa) was added to the obtained products to degrade the free RNA. To synthesize dscDNA, anchored random primers were added and incubated 75°C for 5 min and then on ice for 5 min for denaturation. Then, 1 μL of Klenow fragment (TaKaRa), 1 μL of 10 mM dNTPs (TaKaRa), 2 μL of 10 × Klenow buffer (TaKaRa), and 6 μL of dd H_2_O (TaKaRa) were added and the samples were incubated at 37°C for 60 min, followed by 75°C for 10 min. Then, 0.5 μL of exonuclease I (TaKaRa), 1 μL of shrimp alkaline phosphatase (SAP, TaKaRa, Dalian, China), 5 μL of 10 × phosphatase buffer (TaKaRa), and 24 μL of DEPC H_2_O (TaKaRa) were added and incubated at 37°C for 60 min followed by 75°C for 10 min to eliminate the phosphates and the free single-strand nucleic acid in the dscDNA reaction.

### Sequence-independent single-primer amplification (SISPA) and purification of PCR products

To increase the quantity of the viral nucleic acids, SISPA was applied to amplify the dscDNA. The 50 μL reaction comprised 10 μL of dscDNA, 2 μL of barcode primer (Table 2), 1 μL of AccuPrime Taq DNA polymerase (Invitrogen), 5 μL of 10 × AccuPrime buffer I (Invitrogen), and 32 μL dd H_2_O (TaKaRa). The PCR condition comprised 95°C for 3 min; 40 circles of 95°C for 20 s, 54°C for 20 s, and 68°C for 70 s; and a final extension at 68°C for 7 min. The obtained PCR products were purified using a PCR purification kit (QIAGEN, Hilden, Germany) and eluted in 30 μL of TE buffer (100 mM Tris-HCl, 10 mM EDTA, pH 8.0) (Promega, Madison, USA).

### Metaviral sequencing

The purified PCR products from the three samples were sent to the Wuhan Genome Institute (BGI, Shenzhen, China) for Illumina sequencing. Briefly, to obtain ~180 bp DNA fragments, the purified PCR products were ultrasonicated, and dATP and Klenow fragment were added to produce 3′-dA overhangs. To establish genomic DNA libraries, DNA fragments were bound to Illumina adaptors and amplified using PCR with adaptor primers. Amplicons were ligated to flow cells to which fluorescently labeled dNTPs were added. DNA sequences were identified by using the sequencing-by-synthesis method (SBS, Illumina). Base calling was monitored by the program GAPipeline (BGI), with default settings. No-calling reads and adaptor sequences were eliminated. The remaining sequences were assembled into contigs using SOAPdenovo software (BGI, Shenzhen, China). Contigs and sequences longer than 100 bp were defined as significant data for further *in silico* analysis.

### Computational analysis

The contigs and sequences were aligned using the blastx and blastn with the nonredundant and viral reference sequences in the GenBank database. (https://www.ncbi.nlm.nih.gov/genbank/) BLAST hits with an *E*-value ≤ 10e^−5^ were considered significant. After eliminating the bacterial and eukaryotic sequences, we analyzed the virus-like sequences.

### Identification of detected viruses

Based on the alignment outcomes of the viral contigs and the match position of the viral contigs with the corresponding viruses in GenBank, specific primers were designed and synthesized to identify the detected viruses. Viral nucleic acids were extracted by Bioer Technology (Hangzhou, China) and amplified using the designed primers and a PCR Master Mix (Tiangen, Beijing, China). To avoid false positives in Illumina sequencing, viral identification was performed three times in an independent manner.

### Phylogenetic analysis

The obtained PCR products were sequenced, and the sequences were aligned against sequences of the representative viruses using CLUSTAL W version 2.0. (Accession numbers are shown in the phylogenetic trees). To achieve better accuracy in phylogenetic analysis, neighbor-joining phylogenetic trees were produced using MEGA 7 with 1,000 bootstrap replicates (Wang et al., 2012; Li, 2015).

### Cell culture

The hamster cell line BHK-21 (conserved in our laboratory) was employed in this study. BHK-21 cells were cultured in Dulbecco's modified Eagle's medium (DMEM) (HyClone, Logan, UT, USA) with 10% fetal bovine serum (FBS) (HyClone) and 1% penicillin and streptomycin (Pen Strep) (HyClone), and incubated at 37°C in 5% CO_2_.

### Isolation of viruses

Viral isolation was conducted with the positive ground mosquito supernatants. Briefly, the ground mosquito supernatants were diluted seven-fold with DMEM containing 2% FBS and cultured with BHK-21 cells for 5–7 days. Cultures were examined daily for evidence of a virally induced cytopathic effect (CPE). Cultures without a CPE were blind-passaged three times.

### Identification of the antiviral effect using an indirect immunofluorescence assay (IFA)

BHK-21 cells were seeded in 12-well plates, and after 24 h of culture, they were rinsed with phosphate-buffered saline (PBS) (Solarbio, Beijing, China) and fixed using methanol (Solarbio) for 10 min at room temperature. The cells were washed three times with PBS and then incubated in blocking buffer [3% bovine serum albumin (BSA) (BOSTER, Beijing, China) in Tris-buffered saline (Solarbio) and Tween20 (Solarbio)] for 1 h at room temperature. The cells were then incubated with an anti-E monoclonal antibody (Abcam, Cambridge, UK) (1:20) in PBS with 3% BSA overnight at 4°C. After the cells were washed three times in PBS, they were incubated with a fluorescently labeled secondary antibody—fluorescein isothiocyanate-conjugated goat antimouse antibody (ZSGB-Bio, Beijing, China)—in the dark at 37°C for 1 h. The nuclei were washed with PBS three times and stained with 4,6-diamidino-2-phenylindole (DAPI) (Solarbio). Slides were imaged under a fluorescence microscope (Olympus, Tokyo, Japan).

### Observation by negative-stain electron microscopy

BHK-21 cells with suspected JEV-induced CPE were collected by repeated freezing and thawing three times, followed by centrifugation at 10,000 × g for 15 min. The obtained virus suspension was centrifuged at 60,000 × g for 4 h and the supernatant was removed gently and discarded. Subsequently, the viral precipitate was resuspended with an isometric mixture of 6.1% (v/v) pH 7.2 glutaraldehyde (HEDEBIO, Beijing, China) fixative and DMEM, 25 μL of which were added to the copper grid. After desiccation, one drop of 3% phosphotungstic acid (JINDU, Shanghai, China) was added for negative staining. Before observation under an electron microscope (FEI, Hillsboro, USA), the grid was placed in an incubator (SANYO, Osaka, Japan) at 37°C for desiccation.

### Amplification and phylogenetic analysis of JEV complete genome

The JEV strain was isolated, and then the complete sequence was amplified using RT-PCR. The designed primers are displayed in Table 3. The complete JEV genome was assembled by PCR products using SeqMan version 7.1 (DNAStar, Madison, USA) with the default value. Subsequently, phylogenetic analysis was performed based on the complete sequence of JEV with other representative JEV strains reported in China and neighboring countries.

**Table 3:** Primer pairs used for the complete JEV genome.

**Primer name**	**Primers (5′-3′)**	**Product (bp)**
JEV-1F	AGAAGTTTATCTGTGTGAACTTCTT	379
JEV-1R	CTTGGTCGGGGCTAATGCTGTA	
JEV-2F	TGGCTTAGTATCGTTGAGAAGAATCG	679
JEV-2R	GTAGACTTCCTGATTGTCGCACCA	
JEV-3F	ATGAAGTTGTCGAATTTCCAG	527
JEV-3R	CCCATTCCCAGACAATTAAAA	
JEV-4F	TTTAATTGTCTGGGAATGGGC	1500
JEV-4R	TCAATGGCACATCCAGTGTCA	
JEV-5F	GACACTGGATGTGCCATTGAC	1265
JEV-5R	CTGTTGGCCTCTGCGAAAGCA	
JEV-6F	GCTTTCGCAGAGGCCAACAGT	650
JEV-6R	AACTCAGTAGCTGGCCACCCT	
JEV-7F	GGGTGGCCAGCTACTGAGTTT	413
JEV-7R	GTGTCCCAAAACACGCCCCCT	
JEV-8F	AAAACAACAAAAAGAGGGGGCG	1907
JEV-8R	ATGCGACCGAGCACCTCTATGA	
JEV-9F	TCAGCCGTTAGCTTCATAGAG	826
JEV-9R	ATGTACCCATAGTGAAGTGTC	
JEV-10F	ACACTTCACTATGGGTACATG	431
JEV-10R	GTCCTGCCCCCAGGCCTTCCC	
JEV-11F	GGAAGGCCTGGGGGCAGGACG	2735
JEV-11R	TTCTACCTTAAATCACACTAG	
JEV-12F	GCACGACTGGCAGCAAGTTCCC	1186
JEV-12R	AGATCCTGTGTTCTTCCTCACCAC	

### Detection of the viral titer of different passages

The viral titers of different passages of JEV (passage 5, passage 10, passage 20, and passage 30) were evaluated in BHK-21 cells in triplicate, with DMEM as a control. Cells were incubated with serial ten-fold dilutions of JEV or DMEM for 120 h at 37°C. The viral titer TCID_50_ (50% tissue culture infective dose) was calculated by using the Reed–Muench method (Krah, 1991), according to the cytopathic effect (CPE) in cells caused by viruses. It was evaluated based on the changes in cell culture morphology under a light microscope (OLYMPUS, Tokyo, Japan) that were observed at 100 × magnification. The CPE of JEV or DMEM at each dilution was evaluated in eight replicates.

### Variability of envelope (E) genes of different passage viruses

Viral RNA was extracted from the supernatant using an RNA viral kit according to the manufacturer's protocol (Bioer Technology) after a CPE was induced in BHK-21 cells by different passage viruses (P5, P10, P20, and P30). The purified RNA was used as the template for cDNA synthesis using the SuperScript™ III first-strand synthesis system (Invitrogen) with the reverse primer JWR1 5′-AGATCCTGTGTTCTTCCTCACCACCA-3′, according to the manufacturer's instructions. The envelope (E) genes of different passage viruses were amplified using the paired primers shown in Table 4 and sequenced. The alignment of the four E genes was performed using MEGA 7.0.

**Table 4:** Primer pairs used for identification by means of nested PCR.

**Primer name**	**Primers (5′-3′)**	**Product (bp)**
DENV-China/YN2016-1-F1	ACATGGAGGTTGAAATTTG	2062
DENV-China/YN2016-1-R1	GGTTTTCTATGCCTTGTGG	
DENV-China/YN2016-1-F2	GATAGCCATTGCGGTAGCTAGT	1231
DENV-China/YN2016-1-R2	ATGGGAATCGGTTCCTCATGTCCTG	
DENV-China/YN2016-2-F1	TGGCAGAAACACAGCATGGG	1730
DENV-China/YN2016-2-R1	GTTGATCTAATTCCACAGAC	
DENV-China/YN2016-2-F2	GTGAACAAGGAAAAAGTGGTTGGGCG	491
DENV-China/YN2016-2-R2	TGCACGTTGTCAATTACAAAAATT	
DENV-China/YN2016-3-F1	GACATGACAATCATTGGGAG	1617
DENV-China/YN2016-3-R1	GAGCATATCTTCAGTGGTC	
DENV-China/YN2016-3-F2	CATCAGCCAG TGAAGCTGTGAAT	939
DENV-China/YN2016-3-R2	ATCCAGCCCCTTGCGAGATTC	
ZIKV-China/YN2016-1-F1	GATGGACTCAGCGAGGTAC	1198
ZIKV-China/YN2016-1-R1	GTCAAGAAGCATTCTTGCTTC	
ZIKV-China/YN2016-1-F2	CGGAGAGAGGGCCAGAAACATTC	694
ZIKV-China/YN2016-1-R2	GGACTTCCACTTCTGTGTCCAT	
ZIKV-China/YN2016-2-F1	CTGGGCCCATGCCTGTCACG	1313
ZIKV-China/YN2016-2-R1	GATAGCTACTATCAGAGTCAG	
ZIKV-China/YN2016-2-F2	GAACCCTAACAAACCTGGAGATG	430
ZIKV-China/YN2016-2-R2	AAGCCGCTCCTCTTTTTCCAGCG	
JEV-China/YN2016-1-F1	TAACAGCCTGTGCCGGAGCC	2001
JEV-China/YN2016-1-R1	TGTCAATGGCGCAGCCAGTGTC	
JEV-China/YN2016-1-F2	CTATTGGTCGCTCCGGCTTACAGT	1500
JEV-China/YN2016-1-R2	TGTCAATGGCGCAGCCAGTGTC	

### Statistical analysis

The data were analyzed using SPSS 17.0 software. Group comparisons for viral titers were carried out by using the *t*-test for independent means. Experiments were performed in triplicate and a minimum of three independent experiments were evaluated. The value of *P* < 0.05 was considered statistically significant.

### Genbank accession numbers

The data from Illumina sequencing have been deposited in the GenBank Sequence Reads Archive under accession numbers SRR7204303 to SRR7204305. The GenBank accession numbers of DENV-China/YN2016-1, DENV-China/YN2016-2, DENV-China/YN2016-3, ZIKV-China/YN2016-1, and ZIKV-China/YN2016-2 sequences are MG751801–MG751805. The GenBank accession numbers for the E gene and the complete genome of JEV-China/YN2016-1 are MG644382 and MH385014.

## Results

### Metaviral sequencing and the virome of mosquitoes

**Figure 2:**
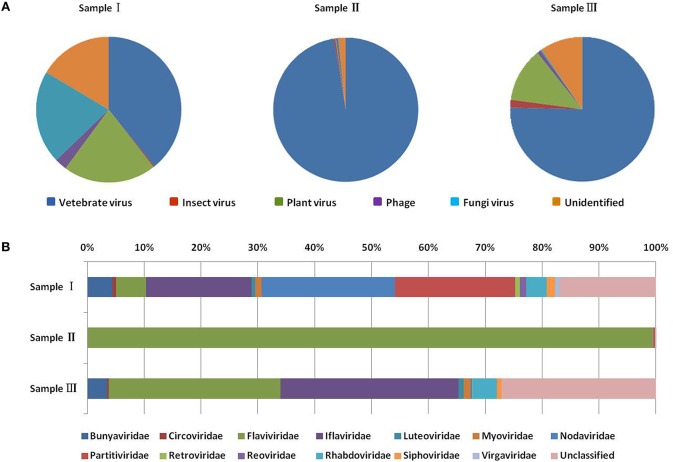
Categories of viral hosts and families of viral sequences in the three mosquito samples. Viral sequences were sorted according to the viral host category **(A)**. The proportions of fungal viruses are too small to be seen in the figure. Viral sequences are classified at the family level **(B)**. Families with <10 reads are not shown. Different host categories and families are indicated by different colors.

To obtain the clean data of the virome of the mosquitoes, contaminating host sequences and barcode DNA were eliminated. A total of 22,964,305 reads were acquired by Illumina sequencing, with averaging read lengths of 186 nt (Table 1). The amount of viral sequences was 17.00, 0.24, and 13.85% in sample I, II, and III respectively and the viruses could be clearly classified (Figure 2). Among them, 87.4% were vertebrate viruses, 5.4% were plant viruses, and 1% were fungal viruses; insect viruses and phages accounted for 0.8 and 0.6%, respectively (Figure 2A). Most viral sequences in Sample I were from *C. tritaeniorhynchus*, the predominant JEV-transmitting vector in China, followed by *A. obturbans*. *A. albopictus* is the predominant ZIKV-transmitting vector. Viral sequences were classified at the family level (Figure 2B), and *Flaviviridae* was the predominant viral family, together with certain other important viral families such as *Circoviridae, Anelloviridae, Parvoviridae*, and *Peribunyaviridea*. Additionally, numerous unclassified viral sequences, presumably belonging to novel viruses not previously identified in mosquitoes, were identified and are worthy of further study.

### PCR verification of the metavirome results

Viral sequences were assembled into contigs using SOAPdenovo. From the 13,645 assembled viral contigs, 32 DENV-like contigs with a read coverage of 184 × (236–1408 nt), 13 ZIKV-like contigs with a read coverage of 34 × (194–652 nt), and 26 JEV-like contigs with a read coverage of 52 × (306–1,564 nt) were obtained. The DENV-like contigs shared 92–98% nt identity with known DENV sequences. The ZIKV-like contigs shared 95–99% nt identity with known ZIKV sequences. The JEV-like contigs shared 96–99% nt identity with known JEV sequences. To confirm the outcome of metavirome sequencing, specific primers were designed and synthesized to amplify the identified viruses (Table 4). The following were the results of PCR identification in mosquito samples.

#### PCR amplification of dengue virus

**Figure 3:**
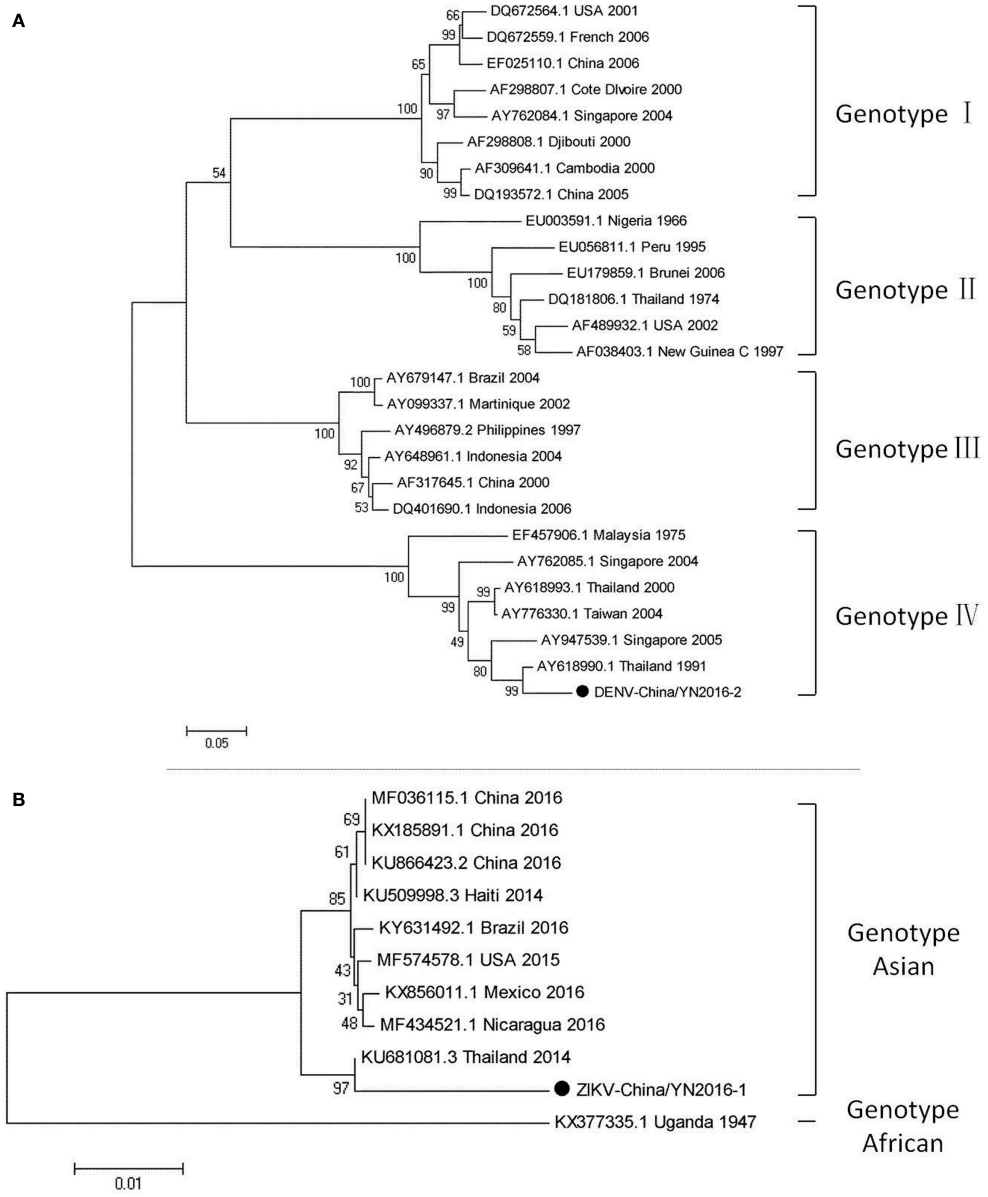
Phylogenetic trees of DENV and ZIKV. Phylogenetic trees based on the E gene of DENV **(A)** and the nonstructural protein NS3 of ZIKV **(B)**. The trees were constructed using the p-distance-based neighbor-joining method in MEGA 7.0 software. Bootstrap values were calculated with 1,000 replicates. Black solid circles indicate the genes identified in this study.

Dengue virus belongs to *Flaviviridae*, and 3,044 cases of dengue were reported in China in 2005–2012, among which 134 cases were reported in Yunnan province. Currently, there is no DENV-5 case reported in China. There have been DENV 1–4 outbreaks in China repeatedly and alternately. However, DENV-1 is predominant. (Xiong and Chen, 2014). Based on the alignment results of viral contigs, viral PCR amplicons from samples II shared a high identity with the dengue virus (DENV) genes, indicating that they belong to insect viral species. The results were verified by nested RT-PCR, which identified a 1,231 nt sequence (DENV-China/YN2016-1) encoding a partial nonstructural protein 4A, 4B, and 5 gene; a 491 nt sequence (DENV-China/YN2016-2) encoding a partial E protein, and a 939 nt sequence (DENV-China/YN2016-3) encoding a partial nonstructural protein 5 gene from DENV. Phylogenetic analysis showed that the newly identified DENV sequences were genotype IV. In addition, DENV-China/YN2016-2 was highly homologous to a DENV sequence from Thailand that was isolated in 1991, sharing 95.3% nt identity. This suggested that the viruses represented a DENV variant (Figure 3A). Interestingly, the partial E gene in DENV-China/YN2016-2 may represent a new genotype IV gene in the DENV variant strain.

#### PCR amplification of Zika virus

The first imported case of ZIKV, pertaining to *Flaviviridae*, was confirmed in China in February 2016 (Liu et al., 2016; Zhang et al., 2016). A total of 28 cases were reported in Zhejiang province, China in September 2016 (Deng et al., 2016; Li et al., 2016). In addition, 19 cases were reported in Guangdong province, China in 2016 (Sun et al., 2017). In line with the alignment results of viral contigs, viral PCR amplicons from sample II shared a high identity with ZIKV, also belonging to insect viral species. These results were also confirmed by means of nested RT-PCR, which identified 694 and 470 nt sequences, both encoding partial nonstructural protein 3 from ZIKV. These sequences were named ZIKV-China/YN2016-1 and ZIKV-China/YN2016-2, respectively. Phylogenetic analysis showed that the newly identified Zika virus sequence ZIKV-China/YN2016-1 was from an Asian genotype. The nucleotide identity analysis revealed that it shared the highest nucleotide sequence identity (97.1%) with a ZIKV sequence from Thailand that was isolated in 2004 (Figure 3B).

#### PCR amplification of JEV

**Figure 4:**
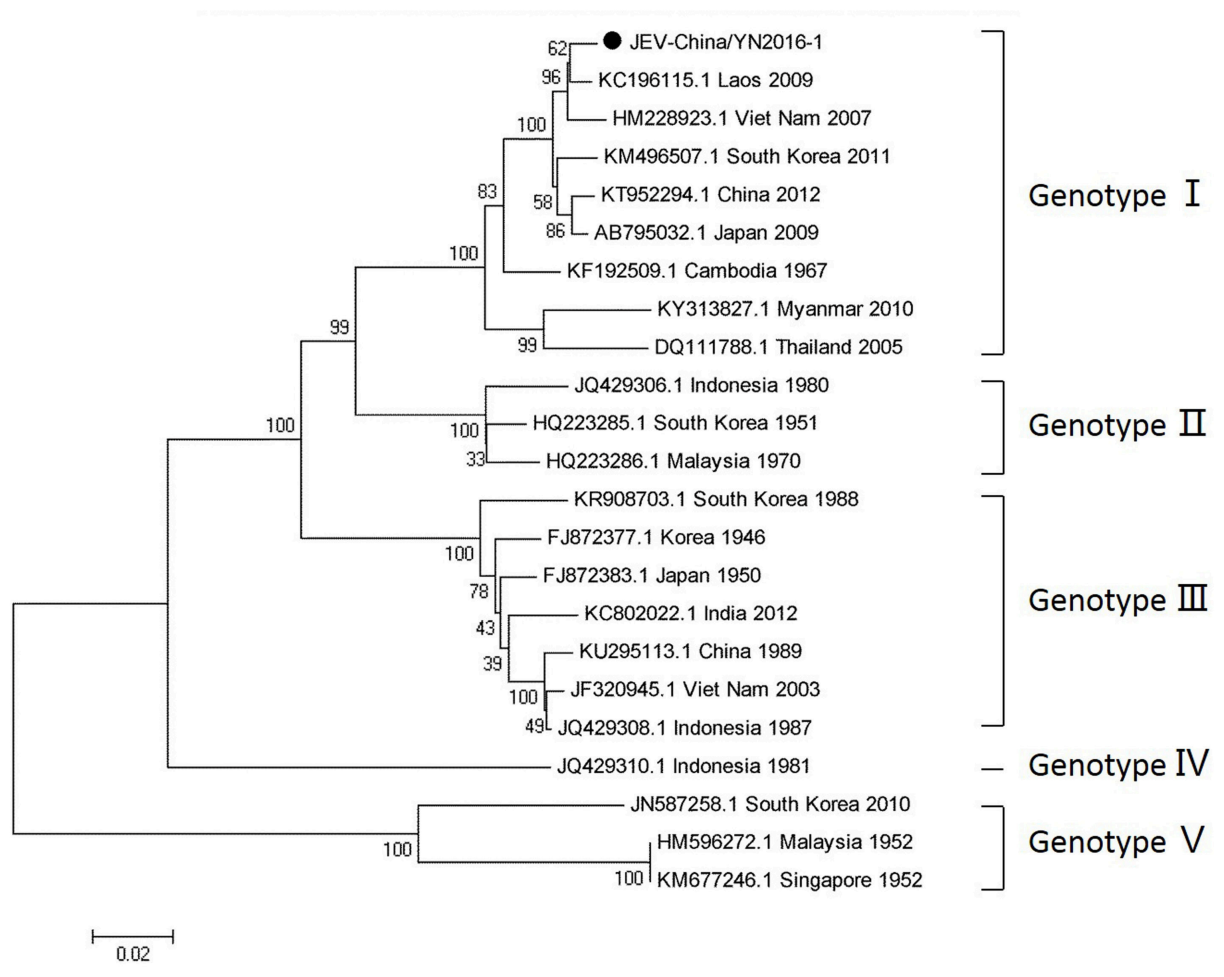
Phylogenetic trees of the JEV E gene. Phylogenetic trees based on the E gene of JEV. The trees were constructed using the p-distance-based neighbor-joining method in MEGA 7.0 software. Bootstrap values were calculated with 1,000 replicates. Black solid circles indicate the genes identified in this study.

Since 1951, 33,900 cases of JEV, which belongs to *Flaviviridae*, were reported in China. The most obvious feature for the territory distribution of JE in China in the 1970s or 2010 is that a small number of cases were reported in west and north China, whereas a high number of cases occurred in east and southwest China. Yunnan province is still a highly epidemic area for JEV. First reported in 1949, JEV has existed in China for more than 60 years. The prevalence of JEV is genotype I and III in China, and genotype I is predominant in Yunnan province (Zheng et al., 2012). According to the alignment results of viral contigs, viral PCR amplicons from sample I shared a high identity with JEV genes, indicating that they belong to insect viral species. The results were verified by means of nested RT-PCR, which identified a 1,500 nt sequence (JEV-China/YN2016-1) encoding a complete E protein. Phylogenetic analysis showed that the newly identified JEV sequence, JEV-China/YN2016-1, was genotype I (Figure 4). Nucleotide identity analysis revealed that it shared the highest nucleotide sequence identity (98.8%) with a JEV sequence from Laos that was isolated in 2009.

### Viral identification of JEV and phylogenetic analysis of its complete genome

**Figure 5:**
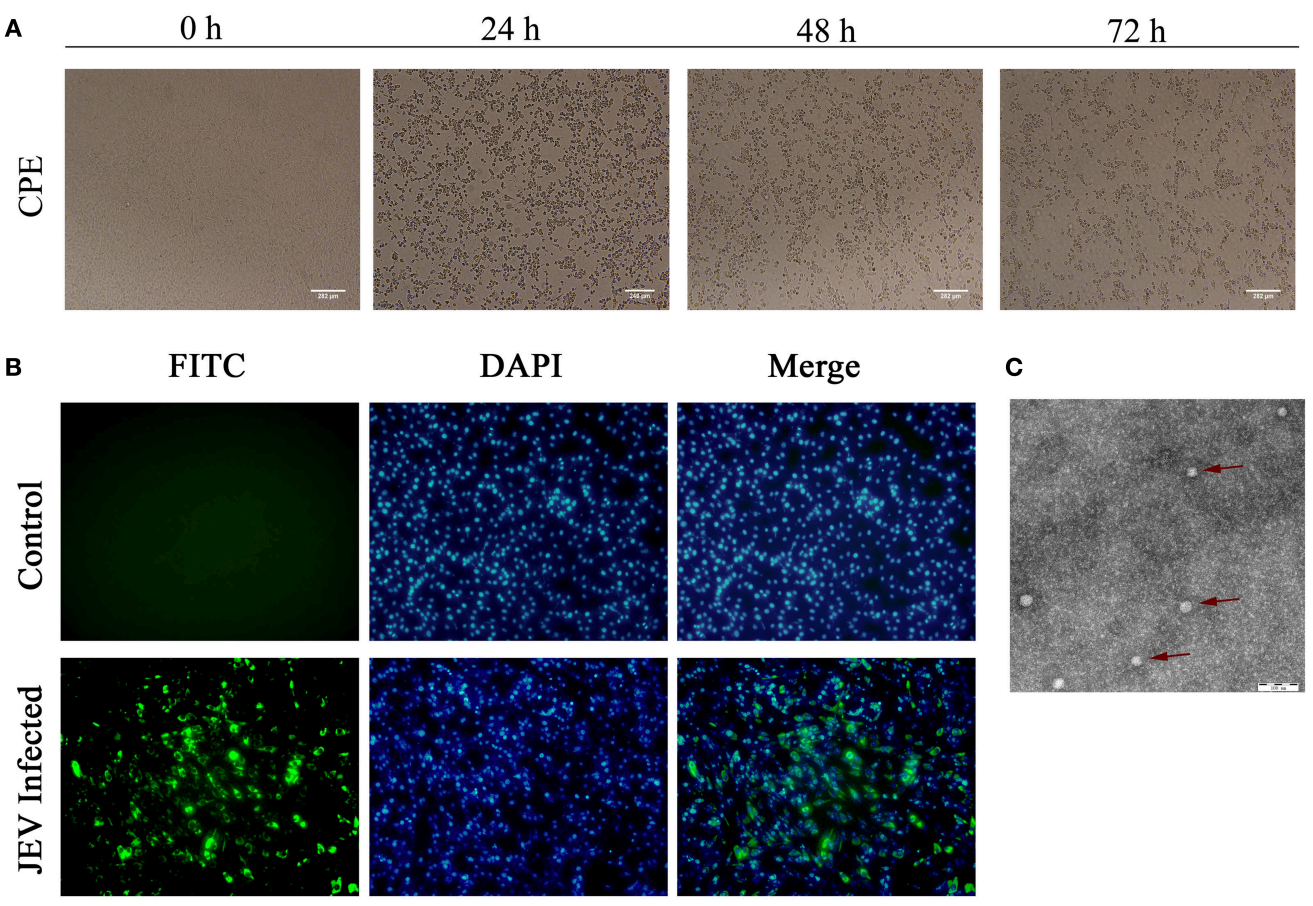
Identification of *JEV*-China/YN2016-1 isolated in Ximeng county of Yunnan province by CPE, IFA, and negative-stain electron microscopy. The CPE of BHK-21 cells infected with *JEV*-China/YN2016-1 at 24, 48, and 72 h **(A)**. The indirect immunofluorescence assay (IFA) of the strain *JEV*-China/YN2016-1 using an anti-E monoclonal antibody (Abcam, Cambridge, UK) and FITC-conjugated goat antimouse antibody (ZSGB-Bio, Beijing, China) **(B)**. Negative-stain electron microscopy of *JEV*-China/YN2016-1 particles **(C)**.

**Figure 6:**
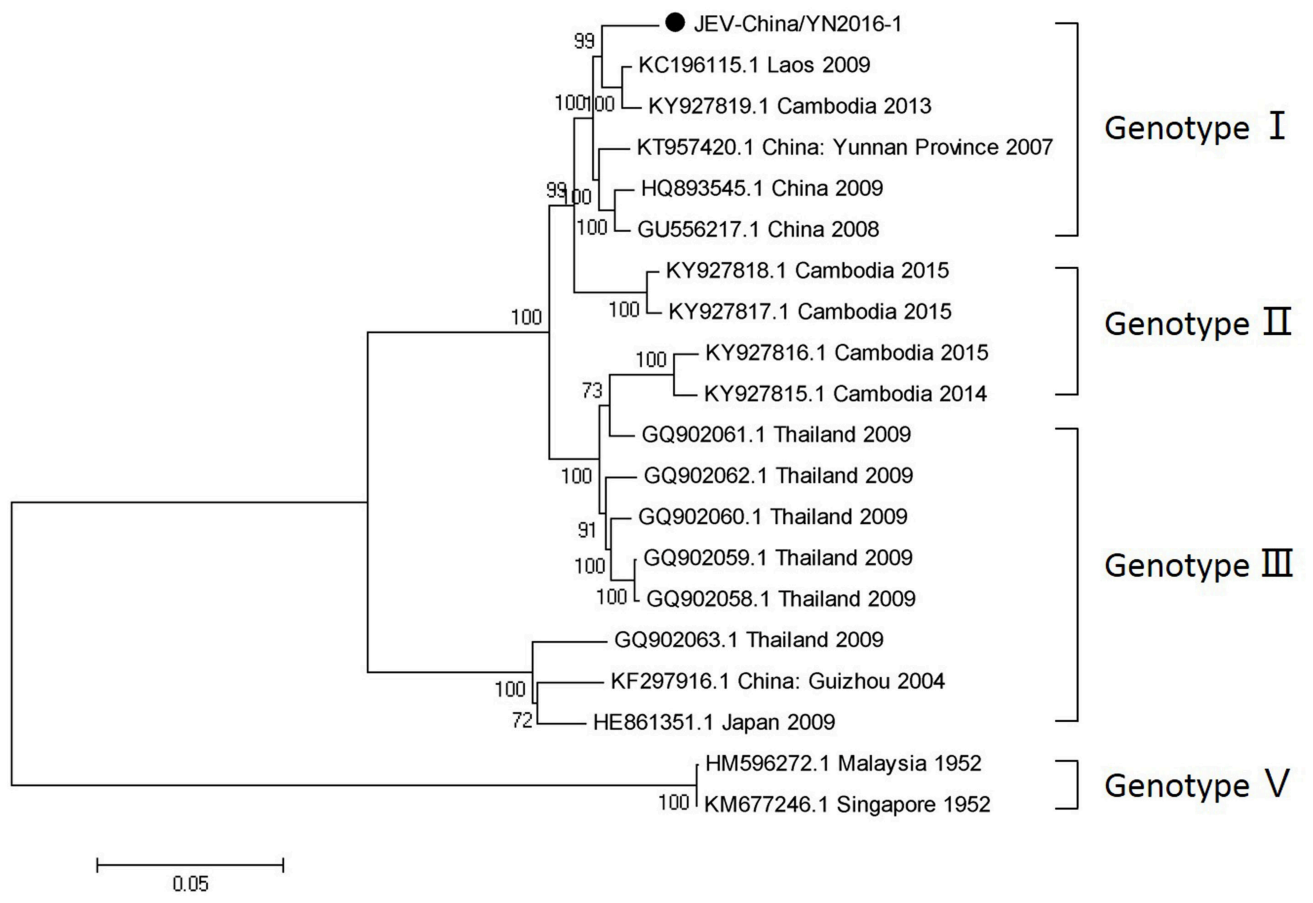
Phylogenetic trees of the complete JEV genome. Phylogenetic trees based on the complete genome of JEV. The trees were constructed using the p-distance-based neighbor-joining method in MEGA 7.0 software. Bootstrap values were calculated with 1,000 replicates. Black solid circles indicate the complete genome of JEV isolated in this study.

After inoculation into BHK-21 cells, the obtained JEV strain induced an obvious CPE. Monolayer cells appeared rounded, with bulging, aggregation, shedding, and incomplete destruction of a single loose layer, with gaps between cells. The severity of the CPE was time-dependent. JEV was propagated in BHK-21 cells for 24 h. The levels of intracellular virus were measured by IFA using an E-specific monoclonal antibody. As expected, mock-infected cells showed no E expression, while JEV-infected cells were positive for E expression. Viral particles were observed using negative-stain electron microscopy. The particles were rounded with a diameter of ~30–40 nm. Moreover, there were tiny protrusions on the surface, similar to JEV particles (Figure 5). The phylogenetic analysis of the complete JEV genome showed that JEV-China/YN2016-1 still shared the highest nucleotide sequence identity (97.6%) with a JEV sequence from Laos that was isolated in 2009 (Figure 6).

### Viral titer of JEV-China/YN2016-1 of different passage viruses

In BHK-21 cells, the averages of viral titers of JEV-China/YN2016-1 of different passage viruses were 2.82 × 10^4^ TCID_50_/0.1 mL (P5), 7.59 × 10^5^ TCID_50_/0.1 mL (P10), 3.16 × 10^5^ TCID_50_/0.1 mL (P20), and 2.14 × 10^4^ TCID_50_/0.1 mL (P30). This showed that there was variability in viral titers. To test the evolution of viruses, a JEV strain with GenBank accession number JQ086762.1 was set as the control. Its averages of viral titers were 3.73 × 10^5^ TCID_50_/0.1 mL (P5), 4.26 × 10^5^ TCID_50_/0.1 mL (P10), 3.87 × 10^5^ TCID_50_/0.1 mL (P20), and 4.14 × 10^5^ TCID_50_/0.1 mL (P30). This indicated that the viral titers of the control were stable.

### Variability of JEV-China/YN2016-1 E gene of different passage viruses

To avoid enzyme errors or sequencing errors, DNA polymerase Ex Taq (TaKaRa, Dalian, China), Fly DNA Polymerase (Transgen Biotech, Beijing, China), and Taq Master Mix (Tiangen, Beijing, China) were used three times to amplify the E gene of the JEV strain and the control at different viral passages. The PCR products of each viral passage were all sequenced, with consistent results in repeated trials. The JEV-China/YN2016-1 E genes of different passage viruses (P5, P10, P20, and P30) were aligned using MegAlign version 7.1 (DNAStar, Madison, USA) with the default value. Nucleotide identity analysis showed that P5 shared 99.1–99.2% nucleotide identity with the other passage viruses (P10, P20, and P30). P10 possessed 100% nucleotide identity with P20, and they both had 99.9% nucleotide identity with P30. By nucleotide alignment, 13 nucleotide sites in the E gene appeared with variation. Amino acid identity analysis showed that P5 shared 99.8% identity with P10 and P20, which shared 100% identity with each other. Interestingly, P5 and P30 had the same amino acid sequences, with no variation. By means of amino acid sequences alignment against those of P10 and P20, one amino acid site (position 138 aa) of E proteins was mutated, with a change of amino acid K to E.

## Discussion

Mosquitoes are the intermediate hosts of numerous viruses that infect humans, animals, insects, plants, and other species, and play a significant role in the prevalence of many infectious diseases (Zhang et al., 2013; Fisher et al., 2015; Shi et al., 2015). Before 2007, virus identification by traditional virological methods required several decades and involved laboratories worldwide (Krah, 1991). Compared with the traditional method, metagenomic sequencing by Illumina sequencing combined with a high throughput analysis is more efficient, resulting in the identification of many viruses from different species in recent years by several laboratories (Wilson et al., 2015). Moreover, metagenomic sequencing has revealed the high abundance of viruses in mosquitoes (Bolling et al., 2015; Fauver et al., 2016; Shi et al., 2017). Metagenomic sequencing has also contributed to the discovery of novel viruses and their identification and characterization (Carissimo et al., 2016; Frey et al., 2016). In the present study, more than 50 viral families were detected by Illumina sequencing in mosquito samples. However, there was a considerable difference in the percentage of viral sequences obtained from the three mosquito groups. It could be related to the biology of the vectors (Atoni et al., 2018; Xia et al., 2018; Zakrzewski et al., 2018). Still, new findings of viral distribution and evolution in Yunnan province were obtained.

RT-PCR and nested-PCR were used to confirm the results of Illumina sequencing. The detection of DENV in the mosquito samples showed that the virus still exists in Yunnan province, and many strains of DENV have been uncovered by previous studies (Guo et al., 2013; Shi et al., 2014; Zhang et al., 2014; Wang et al., 2016). However, the detection of new DENV strains is important, as it suggests a new prevalence of DENV in this area. Moreover, the identification of the Zika virus genes is significant, suggesting that increased environmental surveillance is required because of its strong infectivity to humans, especially pregnant women and infants. Meanwhile, the presence of unidentified viruses in the Illumina sequencing results may ascribed to inadequate mosquito sampling and limited collection sites.

ZIKV and DENV were detected simultaneously, indicating that these viruses co-circulate in Yunnan province and suggesting that co-infection by the viruses is possible. Zika virus pathogenesis can be enhanced by preexisting dengue virus (Bardina et al., 2017). The simultaneous detection of the genes of two viruses may represent a new challenge to the control of ZIKV. We performed the isolation of DENV and ZIKV viruses in Vero and C6/36 cells besides BHK-21 cells as well as the suckling-mouse brain was also used in viral isolation. It was unfortunate that we could not isolate DENV and ZIKV.

The detection and isolation of JEV indicated that the virus still exists in Yunnan province. Analyzing the variant sites in the E gene and the consequences for the sequence of the encoded protein, we discovered that amino acid 138 in the E proteins of viruses at P5 and P30 is K (lysine). However, amino acid 138 in the E proteins of viruses at P10 and P20 is E (glutamic). Lysine at position 138 in the E protein is an attenuated viral site (Ni et al., 1994). The results showed that JEV-China/YN2016-1 has undergone mutations when passaged in BHK-21 cells, suggesting the possibility of increased threat to human health induced by JEV variation. The phylogenetic analysis based on the complete genome and the E gene of JEV-China/YN2016-1 showed that it shared the highest nucleotide sequence identity with a JEV sequence from Laos that was isolated in 2009, indicating the possible threat of JEV transboundary transmission.

This is the first report of the detection of Zika virus genes in Yunnan province, China. The newly identified DENV-China/YN2016-2 is a novel genotype IV gene in the DENV mutant strain. Metagenomic analysis of the viromes of mosquitoes in Yunnan province, combined with nested PCR, revealed that the distribution of viruses is dependent on both the mosquito species and the geographical location. The present work explored only a small portion of the virome of mosquitoes prevailing in this region of China. A much larger study on mosquitoes in other provinces in China and from other countries is needed to assess the diversity of mosquito-borne viruses. In conclusion, our findings provide a useful insight into the viral isolation and the characterization of Zika and dengue viruses and could hence be applied to larger studies in the future.

## Author contributions

PX, HJL, and NJ conceived and designed the experiments. PX, JH, CL, SW, YL, MW, and NJ performed the experiments. PX, HL, JR, and NJ analyzed the data. YZ, XG, HZ, HJL, and MT contributed reagents, materials, and analysis tools. PX and NJ wrote the paper. All authors read and approved the final version of the manuscript.

### Conflict of interest statement

The authors declare that the research was conducted in the absence of any commercial or financial relationships that could be construed as a potential conflict of interest.
